# Capsaicin Alleviates the Deteriorative Mitochondrial Function by Upregulating 14-3-3*η* in Anoxic or Anoxic/Reoxygenated Cardiomyocytes

**DOI:** 10.1155/2020/1750289

**Published:** 2020-03-03

**Authors:** Yang Qiao, Tianhong Hu, Bin Yang, Hongwei Li, Tianpeng Chen, Dong Yin, Huan He, Ming He

**Affiliations:** ^1^Jiangxi Provincial Institute of Hypertension, The First Affiliated Hospital of Nanchang University, Nanchang 330006, China; ^2^Jiangxi Provincial Key Laboratory of Basic Pharmacology, Nanchang University School of Pharmaceutical Science, Nanchang 330006, China; ^3^Jiangxi Provincial Key Laboratory of Molecular Medicine, The Second Affiliated Hospital of Nanchang University, Nanchang 330006, China

## Abstract

Reactive oxygen species (ROS) are byproducts of a defective electron transport chain (ETC). The redox couples, GSH/GSSG and NAD^+^/NADH, play an essential role in physiology as internal defenses against excessive ROS generation by facilitating intracellular/mitochondrial (mt) redox homeostasis. Anoxia alone and anoxia/reoxygenation (A/R) are dissimilar pathological processes. In this study, we measured the impact of capsaicin (Cap) on these pathological processes using a primary cultured neonatal rat cardiomyocyte in vitro model. The results showed that overproduction of ROS was tightly associated with disturbed GSH/GSSG and NAD^+^/NADH suppressed mt complex I and III activities, decreased oxygen consumption rates, and elevated extracellular acidification rates. During anoxia or A/R period, these indices interact with each other causing the mitochondrial function to worsen. Cap protected cardiomyocytes against the different stages of A/R injury by rescuing NAD^+^/NADH, GSH/GSSG, and mt complex I/III activities and cellular energy metabolism. Importantly, Cap-mediated upregulation of 14-3-3*η*, a protective phosphoserine-binding protein in cardiomyocytes, ameliorated mt function caused by a disruptive redox status and an impaired ETC. In conclusion, redox pair, mt complex I/III, and metabolic equilibrium were significantly different in anoxia alone and A/R injury; Cap through upregulating 14-3-3*η* plays a protection against the above injury in cardiomyocyte.

## 1. Introduction

Aging, hypoxia, ischemia, and ischemia/reperfusion (I/R) are the primary causes of cardiovascular disease [1, 2]. Ischemia (anoxia) and I/R (anoxia/reoxygenation, A/R) injury can be generally divided into two stages: anoxia alone and A/R [3]. Reactive oxygen species (ROS) participate in several pathophysiologic processes (e.g., cellular damage, aging, and apoptosis) during the above injury [4–6]. This injury causes excessive ROS generation, resulting in severe myocardial damage [3–9]. However, ignoring the close relationship between redox balance and ROS in cellular pathological conditions often prevents clinical trials from recognizing the significance of decreasing disease risk and progression.

Glutathione (GSH) converts into glutathione disulfide (GSSG) under oxidative stress. GSH/GSSG ratio sustains the redox homeostasis in cardiomyocyte by decreasing elevated ROS generation [10–12]. An equilibrium between nicotine adenine dinucleotide (NAD^+^, oxidized) and NADH (reduced) is also an essential regulator of the redox system under the pathologic condition of anoxia or A/R; however, the imbalance of NAD^+^ and NADH can also influence oxygen radical levels at the site of complex I on the mitochondrial (mt) electron transport chain (ETC) [13–15]. mt complexes I and III are a major source of ROS in cardiomyocytes [4, 16, 17]. Previous studies documented that decreased complex I/III activities result in excessive ROS accumulation and influence energy metabolism [18–21]. A metabolic disorder is closely associated with the mitochondrial dysfunction of cardiomyocytes during A/R injury [22, 23]. During anoxia, insufficient oxygen supply decreases in oxygen consumption rates (OCR) and adenosine triphosphate (ATP) production inhibiting the ability to meet the demands of energy metabolism and ultimately inducing an irreversible injury on cardiomyocytes [24]. Although oxygen restoration is necessary for salvaging anoxic cell death, it also induces cellular injury due to excessive ROS generation and Ca^2+^ overload [25].

Capsaicin (trans-8-methyl-N-vanillyl-6-nonenamide, C_18_H_27_NO, Cap) is the main active ingredient in plants of the genus Capsicum. Cap has been widely studied as a potential therapeutic agent in diseases such as conjunctivitis, cancer, obesity, and cardiovascular disease [26–29]. Cap is known to have antimicrobial, analgesic, and antioxidant, among other effects [30]. Our recent studies showed that Cap upregulated 14-3-3*η* (a dimeric phospho-serine-binding protein involved in cardiac protection) and SIRT1 (NAD^+^-dependent proteins that act as gatekeepers against oxidative stress and cardiovascular injury) expression in cardiomyocytes in response to A/R injury [7, 8]. The pathologic process of A/R remains unexplored. Cap could have differential modulatory effects on the anoxia and A/R stage.

We performed Cap pretreatments prior to anoxia or A/R injury to test the following: (1) impact of A/R injury on NAD^+^/NADH, GSH/GSSG, mt complexes I/III, and energy metabolism and (2) Cap-mediated effects on redox couples, complex I/III, and energy metabolism.

## 2. Materials and Methods

### 2.1. Reagents

Cap (purity ≥ 98%) was purchased from the National Institutes for Food and Drug Control (Beijing, China). Adenovirus pAD/14-3-3*η*-shRNA was obtained from GeneChem Co., Ltd (Shanghai, China). Antibodies directed against 14-3-3*η*, cytochrome c (cyt C), cleaved caspase-3, Cox4, and *β*-actin were obtained from Cell Signaling Technology (Beverly, MA, USA). Antibodies against NADH dehydrogenase [ubiquinone] 1 beta subcomplex subunit 8 (NDUFB8) and cytochrome b-c1 complex subunit 2 (UQCRC2) were obtained from Abcam (Cambridge, UK). Horseradish peroxidase-conjugated IgG secondary antibody was purchased from Zsbio (Beijing, China).

### 2.2. Primary Cardiomyocyte Culture and Anoxia Alone or Anoxia/Reoxygenation Injury

All experimental protocols were conducted according to the *Guide for the Care and Use of Laboratory Animals* published by the US National Institutes of Health (NIH Publication no. 85-23, revised 1996) and approved by the Ethics Committee of Nanchang University (no. 2019-0036). Cardiomyocytes from 0-3 days old Sprague-Dawley rats (the Animal Center of Nanchang University, Nanchang, China) were prepared as published [7]. Briefly, hearts from neonatal rats were removed and placed in precooling D-Hank's balanced salt solution. The ventricles were digested with 0.1% trypsin and then harvested repeatedly by centrifugation at 600 × g for 5 min. The cells were resuspended in plating medium (80% Dulbecco's Minimal Essential Medium (DMEM), 20% Fetal Bovine Serum (FBS), and 100 U/ml of penicillin and streptomycin) and plated in culture dishes that were incubated 37°C for 30 min to remove nonmyocytes. The suspended cells were plated on 60 mm gelatin-coated culture dishes at 1 × 10^6^ cells per dish and incubated at 37°C in a standard humidity incubator with 95% O_2_ and 5% CO_2_. After 18 hours, cardiomyocytes were washed and plates in fresh medium and incubated for an additional 3 days at 37°C in a standard humidity incubator with 95% O_2_ and 5% CO_2_ before the experiment.

Cardiomyocytes were exposed to three hours of anoxia alone or three hours of anoxia followed by two hours of reoxygenation. Anoxic conditions were generated by incubating the culture plates in an air-tight anoxic chamber placed in a humidified 37°C incubator and passing a mixture of 95% N_2_ and 5% CO_2_. Reoxygenation was provided by placing the cultured plates in a standard humidified 37°C incubator and passing a mixture of 95% O_2_ and 5% CO_2_ [31].

### 2.3. Experimental Grouping and Reagent Treatment

The experimental groups were as follows: during anoxia stage: (1) control group: incubation under normal growth conditions; (2) anoxia group: exposure to anoxic injury; (3) Cap+anoxia group: pretreatment with 10 *μ*M Cap for 36 hours prior to anoxic injury; and (d) pAD/14-3-3*η*-shRNA+Cap+anoxia group: pretreatment with adenovirus pAD/14-3-3*η*-shRNA for 5 hours prior to preconditioning with Cap (36 hours) and anoxic injury.

During the A/R stage, cardiomyocytes were distributed into experimental groups as follows: (a) control group; (b) A/R group: exposure to A/R injury; (c) Cap+A/R group: pretreatment with 10 *μ*M Cap for 36 hours before A/R; and (d) pAD/14-3-3*η*-shRNA+Cap+A/R group: pretreatment with pAD/14-3-3*η*-shRNA for 5 hours prior to preconditioning with Cap (36 hours) and A/R injury.

### 2.4. Measurement of Cell Viability and Biochemical Parameters

Cell viability was measured using a colorimetric assay using the tetrazolium salt WST-8 (TransGen Biotech, Beijing, China). Cardiomyocytes were seeded in 96-well plates at a density of 4 × 10^3^ cells/well. Cells were incubated with 20 *μ*l WST-8 (5 mg/ml) per 100 *μ*l medium for 2 hours at 37°C, and absorbance was measured at 490 nm using a microplate reader (Bio-Rad 680, Hercules, CA, USA). Data was expressed as the ratio between experimental and control groups.

Culture medium after anoxia or A/R treatment was collected to evaluate the activities of lactate dehydrogenase (LDH) and creatine phosphate kinase (CK) using commercially available assay kits (Jiancheng, Nanjing, China) according to the manufacturer's instructions [7].

### 2.5. Preparation of Mitochondrial Fractions and Assessment of NAD^+^/NADH and GSH/GSSG Level

Mitochondrial fractions of cardiomyocytes were prepared using the mitochondria isolation kit (Thermo Fisher, USA). Cells were harvested and centrifuged at 700 × g for 5 min, with the addition of 800 *μ*l ice-cold reagent A and 10 *μ*l precooling reagent B, and incubated for 5 min on ice. Following this, 800 *μ*l of reagent C was added and incubated for 10 min at 4°C. The sample was then centrifuged at 700 × g for 10 min to remove the undissolved protein and debris. The supernatant was collected and centrifuged at 12000 × g for 15 min at 4°C. Then, removed the supernatant and washed the pellet (mitochondria) in 500 *μ*l of reagent C and centrifuged at 12000 × g for 5 min at 4°C. The final pellet was resuspended in lysis buffer containing a protease inhibitor, and the homogenate was designated as the mitochondrial fraction.

NAD^+^, NADH, and NAD^+^/NADH ratios of the mitochondrial fraction were measured using the NAD^+^/NADH Quantification Kit (Sigma-Aldrich, St. Louis, MO, USA). GSH, GSSG, and GSH/GSSG ratios were examined using the GSH and GSSG Assay Kit (Beyotime, Shanghai, China) consistent with the manufacturer's instructions.

### 2.6. Measurement of OCR and ECAR

OCR and extracellular acidification rate (ECAR) were assayed using commercially available assay kits by the Seahorse XFe^24^ Extracellular Flux analyzer (Agilent Technologies, Santa Clara, CA, USA) [32]. Cardiomyocytes were seeded in Seahorse XF Cell Culture Microplate at a density of 4 × 10^3^ cells/well in DMEM supplemented with 10% (*v*/*v*) FBS. A sensor cartridge was added to Seahorse XF Calibrant solution and incubated at 37°C in a non-CO_2_ incubator overnight. Cells were incubated with XF Base Medium (Agilent Technologies) at 37°C in a non-CO_2_ incubator for 45 min prior to the assay. OCR values were assayed under basal/resting conditions and after adding oligomycin, FCCP, rotenone, and antimycin A. Meanwhile, ECAR was measured under basal conditions and with glucose, oligomycin, and 2-DG. The results of OCR and ECAR were calculated from Wave.

### 2.7. Flow Cytometry Assay

ROS levels were assessed with oxidation-sensitive fluorescent probe DCFH-DA (Beyotime, Shanghai, China) [7]. Cells were harvested after treatment as described in Section 2.3 and incubated with DCFH-DA at 37°C for 30 min in darkness. The cells were then centrifuged, washed with ice-cold 1x phosphate-buffered saline (PBS), and detected (excitation (Ex) = 488 nm, emission (Em) = 525 nm) immediately using Cytomics FC500 flow cytometer (Beckman Coulter, Brea, CA, USA).

Mitochondrial Membrane Potential (MMP) was measured using the fluorescent dye JC-1 (BestBio, Shanghai, China) [7]. In brief, cardiomyocytes were incubated with JC-1 for 30 min at 37°C in darkness, centrifuged, and washed to remove the excess reagents. Fluorescence was assessed using Cytomics FC500 flow cytometer at wavelengths of 530/580 nm (red) and 485/530 nm (green). The ratio of the red to green fluorescence intensity of the cells reflected the level of MMP.

Mitochondrial permeability transition pores (mPTP) were assessed utilizing the fluorescent probe BbcellProbe™ M61 (BestBio, Shanghai, China) [33]. Cells were co-incubated with BbcellProbe™ M61 and quenching agent at 37°C for 15 min in darkness and centrifuged at 600 × g for 5 min followed by washing with Hank's balanced salt solution (HBSS). The fluorescence intensity of the dissociated cells was analyzed by a Cytomics FC500 flow cytometer (Ex = 488 nm; Em = 558 nm).

Cells apoptosis was measured according to a method described previously [7]. Cells were collected and resuspended in 1x Annexin V binding buffer. Cell suspension was incubated with 5 *μ*l Annexin V-FITC and 10 *μ*l PI and detected (Ex = 488 nm, Em = 578 nm) directly using Cytomics FC500 flow cytometer.

### 2.8. Western Blot Analysis

Cardiomyocytes were harvested and lysed with RIPA lysis buffer supplemented with a protease inhibitor (phenylmethanesulfonyl fluoride (PMSF)) and incubated for 30 min at 4°C. Protein extracts were centrifuged at 4°C for 15 min to remove insoluble substances. The protein concentration was measured using a bicinchoninic acid (BCA) protein assay kit (Thermo Fisher, USA). Equal amounts of protein (30 *μ*g) were separated by denaturing sodium dodecyl sulfonate polyacrylamide gel electrophoresis (SDS-PAGE) using a gel apparatus and later transferred to a polyvinylidene fluoride (PVDF) membrane. The membrane was blocked with 5% bull serum albumin, washed, and saturated with primary antibodies (14-3-3*η*, 1 : 1000; cleaved caspase-3, 1 : 1000; cyt C, 1 : 1000; NDUFB8, 1 : 500; UQCRC2, 1 : 500; Cox4, 1 : 1000; and *β*-actin, 1 : 1000) overnight at 4°C and then blotted with horseradish peroxidase- (HRP-) conjugated secondary antibody. Subsequently, the membrane was incubated with an enhanced chemiluminescence substrate for 1 min, and protein bands were visualized and analyzed with the Quantity One software (Bio-Rad, USA).

### 2.9. Terminal Deoxynucleotidyl Transferase-Mediated Nick-End Labeling (TUNEL) Assay

Apoptosis was determined using the DeadEnd™ Colorimetric TUNEL System (Promega, USA) and visualized using a fluorescence microscope (Olympus, Tokyo, Japan). Cardiomyocytes were added to microscope slides and fixed with 4% methanol-free formaldehyde at 25°C for 25 min, washed twice with PBS, and permeabilized with 0.2% Triton X-100 at 25°C for 5 min. After washing with PBS, incubation buffer (equilibration buffer, biotinylated nucleotide mix, and recombinant terminal deoxynucleotidyl transferase) was added, and the sample was covered with a plastic coverslip and incubated at 37°C for 1 hour. Subsequently, the slides were immersed in 2x SSC, blocked with 0.3% H_2_O_2_ for 5 min, and incubated with 100 *μ*l HRP for 30 min. Finally, 100 *μ*l of a diaminobenzidine (DAB) solution was added, and the sample was incubated for 5 min in the dark. Next, the sample was rinsed with deionized water and stained with hematoxylin for 1 min. Microscopic analysis was performed as described [7].

### 2.10. Statistical Analysis

Values were represented as mean ± standard error of mean (SEM) from at least six independent experiments. The significance of biochemical data across each group was tested by one-way ANOVA, and the individual differences were tested by least significant difference (LSD) testing. The results were considered statistically significant at a value of *P* < 0.05.

## 3. Results

### 3.1. Cap Protects Cardiomyocytes against Anoxia Alone or A/R Injury

**Figure 1:**
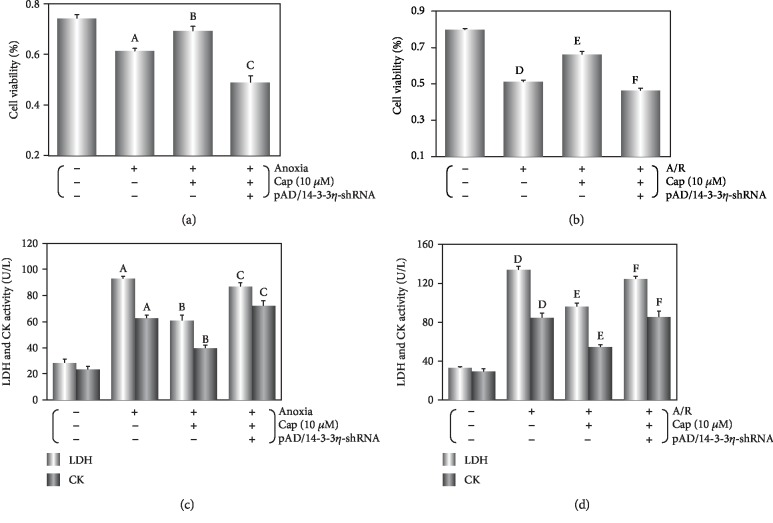
Cap protects cardiomyocytes against anoxia or A/R injury. (a, b) Cell viability of cardiomyocytes. (c, d) LDH and CK activity in culture media. Data are presented as mean ± SEM (*n* = 6). A: *P* < 0.01 vs. control group (anoxia); B: *P* < 0.01 vs. anoxia group; C: *P* < 0.01 vs. Cap+anoxia group; D: *P* < 0.01 vs. control group (A/R); E: *P* < 0.01 vs. A/R group; F: *P* < 0.01 vs. Cap+A/R group.

Cell viability, LDH, and CK activities served as the indicators of cytotoxicity [7]. Following anoxia alone, cell viability decreased, LDH and CK activities increased as compared with the control group (*P* < 0.01), while pretreatment with 10 *μ*M Cap improved cell viability and LDH and CK activities (*P* < 0.05, Figures 1(a) and 1(c)).

Compared with anoxia alone, reoxygenation following anoxia further decreased cell viability (from 61.2 ± 3.2% to 54.1 ± 2.8%, *P* < 0.01, Figures 1(a) and 1(b)) and increased LDH and CK activities (*P* < 0.01, Figures 1(c) and 1(d)), suggesting that reoxygenation stage is an exacerbation period in cardiomyocytes. After treatment with 10 *μ*M Cap, cell viability was similar to the Cap+anoxia group (69.2 ± 3.7% to 71.8 ± 3.6%, *P* > 0.05, Figures 1(a) and 1(b)), and LDH and CK activities were also similar (*P* > 0.05, Figures 1(c) and 1(d)). This could indicate that Cap is able to alleviate cardiomyocyte deterioration. However, the protection of Cap on cardiomyocyte was abrogated by the addition of pAD/14-3-3*η*-shRNA under conditions of anoxia alone or A/R injury (*P* < 0.01, Figure 1).

### 3.2. Cap Upregulates 14-3-3*η* Expression in Cardiomyocytes following Anoxia or A/R Injury

**Figure 2:**
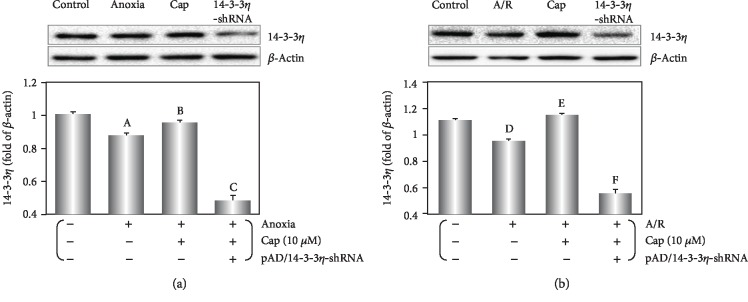
Cap upregulates 14-3-3*η* on cardiomyocytes exposed to anoxia or A/R injury. (a) Western blot and graphic of 14-3-3*η* expression during anoxia treatment. (b) Western blot and graphic of 14-3-3*η* expression during A/R treatment. Data are presented as mean ± SEM (*n* = 6). A: *P* < 0.01 vs. control group (anoxia); B: *P* < 0.01 vs. anoxia group; C: *P* < 0.01 vs. Cap+anoxia group; D: *P* < 0.01 vs. control group (A/R); E: *P* < 0.01 vs. A/R group; F: *P* < 0.01 vs. Cap+A/R group.

14-3-3*η* expression was downregulated by anoxia alone or A/R injury (*P* < 0.01, Figure 2). Following anoxia alone, Cap-pretreated cardiomyocytes slightly increased 14-3-3*η* level (*P* < 0.05, Figure 2(a)), whereas Cap significantly upregulated 14-3-3*η* expression after undergoing AR injury (*P* < 0.01, Figure 2(b)).

### 3.3. Cap Decreases ROS Generation by Maintaining the Redox Balance and Changing Electron Transport in Cardiomyocytes following Anoxia or A/R Injury

**Figure 3:**
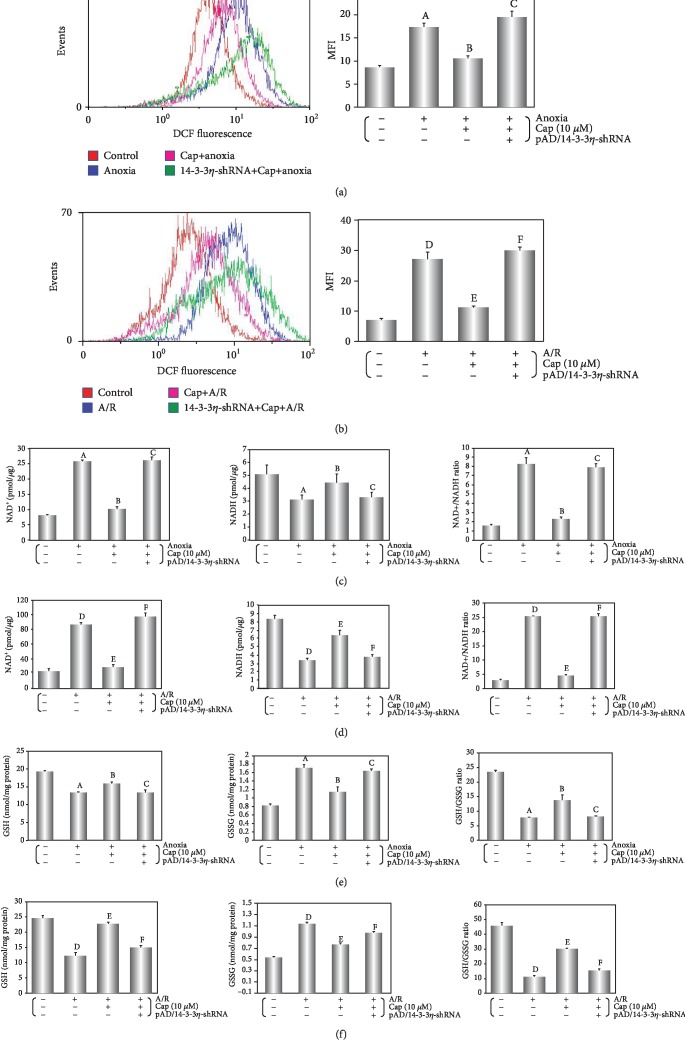
Cap reduces ROS generation by maintaining the redox balance following anoxia or A/R injury. (a, b) Fluorescent probe DCFH-DA indicating ROS level was detected by flow cytometry and column chart of average fluorescence intensity values during A/R exposure. (c, d) Mitochondrial NAD levels in cardiomyocytes after different treatments. Left: histogram of mitochondrial NAD^+^ levels; middle: histogram of mitochondrial NADH levels; right: histogram of mitochondrial NAD^+^/NADH ratio. (e, f) Intracellular glutathione levels of cardiomyocyte after different treatments. Left: histogram of intracellular GSH levels; middle: histogram of intracellular GSSG levels; right: histogram of intracellular GSH/GSSG ratio. Data are presented as mean ± SEM (*n* = 6). A: *P* < 0.01 vs. control group (anoxia); B: *P* < 0.01 vs. anoxia group; C: *P* < 0.01 vs. Cap+anoxia group; D: *P* < 0.01 vs. control group (A/R); E: *P* < 0.01 vs. A/R group; F: *P* < 0.01 vs. Cap+A/R group.

As shown in Figures 3(a) and 3(b), ROS generation increased overall during anoxia or A/R injury when compared with the control group. A/R injury significantly increased ROS generation compared with anoxia alone (from 2.05 (anoxia) to 3.88 (A/R) times, vs. the control group, *P* < 0.01). However, Cap significantly inhibited ROS generation caused by the two treatments (0.61 (anoxia); 0.41 (A/R), vs. the respective injury group, *P* > 0.01). NAD^+^, GSSG, and NAD^+^/NADH increased significantly, while NADH, GSH, and GSH/GSSG decreased significantly after anoxia alone or A/R injury (Figures 3(c)–3(f), *P* > 0.01). The NAD^+^/NADH ratio increased from 5.22 (anoxia) to 9.07 (A/R) (vs. the control group, *P* < 0.01), and the GSH/GSSG ratio decreased from 0.31 (anoxia) to 0.24 (A/R) (vs. the control group, *P* < 0.01). Cap reversed the effects, especially in NAD^+^/NADH and GSH/GSSG ratio (NAD^+^/NADH ratio: 0.28 (anoxia) to 0.18 (A/R), vs. the respective injury group, *P* < 0.01; GSH/GSSG ratio: 1.89 (anoxia) to 2.74 (A/R), vs. the respective injury group, *P* < 0.01). The results showed that A/R injury could activate ROS generation and redox status more than anoxia alone and Cap could rescue the related cellular deterioration.

**Figure 4:**
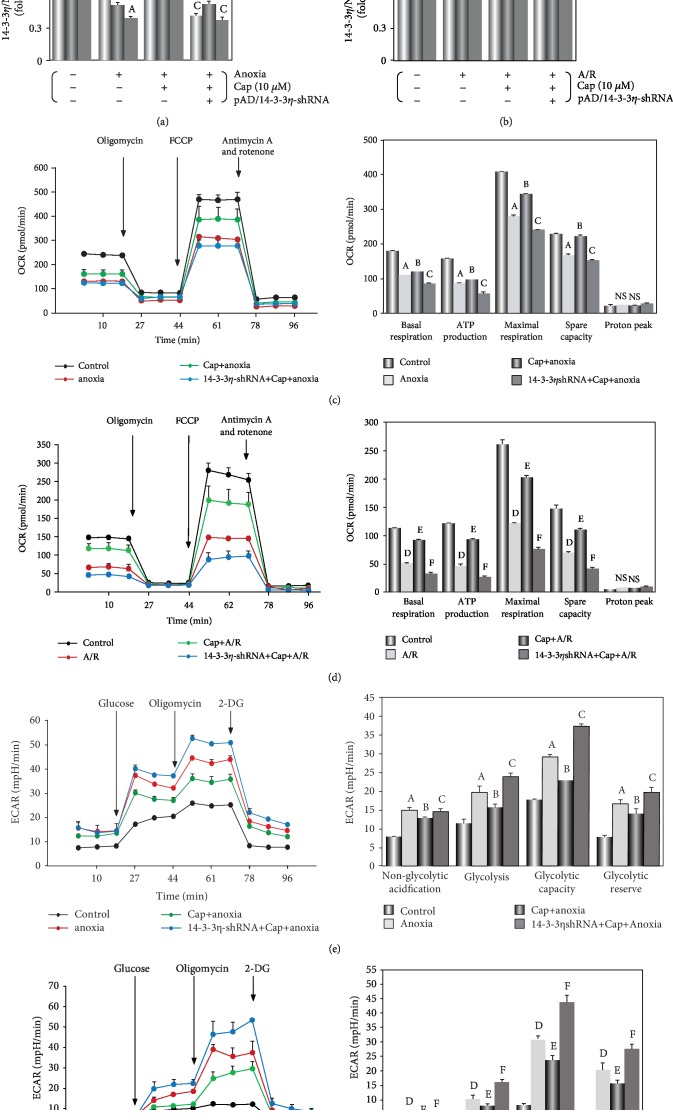
Effects of Cap treatment on mitochondrial bioenergetics and glycolytic activity of cardiomyocytes during A/R injury. (a, b) Western blot and graphic of 14-3-3*η*, NDUFB8, and UQCRC2. (c, d) Effects of Cap on OCR. Cap pretreatment increased mitochondrial respiration following injury. (e, f) Effects of Cap on ECAR. Cap pretreatment decreased lactate accumulation and extracellular acidification. Data are presented as mean ± SEM (*n* = 6). A: *P* < 0.01 vs. control group (anoxia); B: *P* < 0.01 vs. anoxia group; C: *P* < 0.01 vs. Cap+anoxia group; D: *P* < 0.01 vs. control group (A/R); E: *P* < 0.01 vs. A/R group; F: *P* < 0.01 vs. Cap+A/R group.

The levels of NDUFB8 (a subunit of mt complex I) and UQCRC2 (a subunit of mt complex III) were determined by Western blot. NDUFB8 and UQCRC2 levels decreased during the anoxia or A/R exposure (*P* < 0.01, Figures 4(a) and 4(b)), indicating an inhibition of the mitochondrial respiratory chain of cardiomyocyte. This result could partially explain the measured ROS burst and perturbation of mitochondrial redox status including changes in NAD^+^/NADH and GSH/GSSG ratios. The Cap pretreatment significantly increased the mitochondrial complexes of ETC (*P* < 0.01).

Furthermore, OCR measurements showed a significant decrease in basal oxygen consumption, ATP-linked OCR, and spare respiratory capacity over the normoxic control cells (*P* < 0.01, Figures 4(c) and 4(d)). Collectively, these observations indicated that mitochondrial vitality was significantly inhibited following anoxia or A/R injury. ECAR measurements indicated that glycolysis increased significantly after anoxia or A/R injury resulting in lactate accumulation and increased extracellular acidification (*P* < 0.01, Figures 4(e) and 4(f)). As expected, Cap increased OCR and decreased the concentration of extracellular H^+^ in cardiomyocytes during the different types of injury (*P* < 0.01) with a prominent effect in the context of A/R injury. These results corroborate in the data on ROS generation, GSH/GSSG and NAD^+^/NADH ratio, and mt complex I/III activities. The Cap-mediated beneficial effects were abrogated by coincubating with pAD/14-3-3*η*-shRNA (*P* < 0.01).

### 3.4. Cap Improves Mitochondrial Function in Cardiomyocytes Exposed to Anoxia or A/R Injury

**Figure 5:**
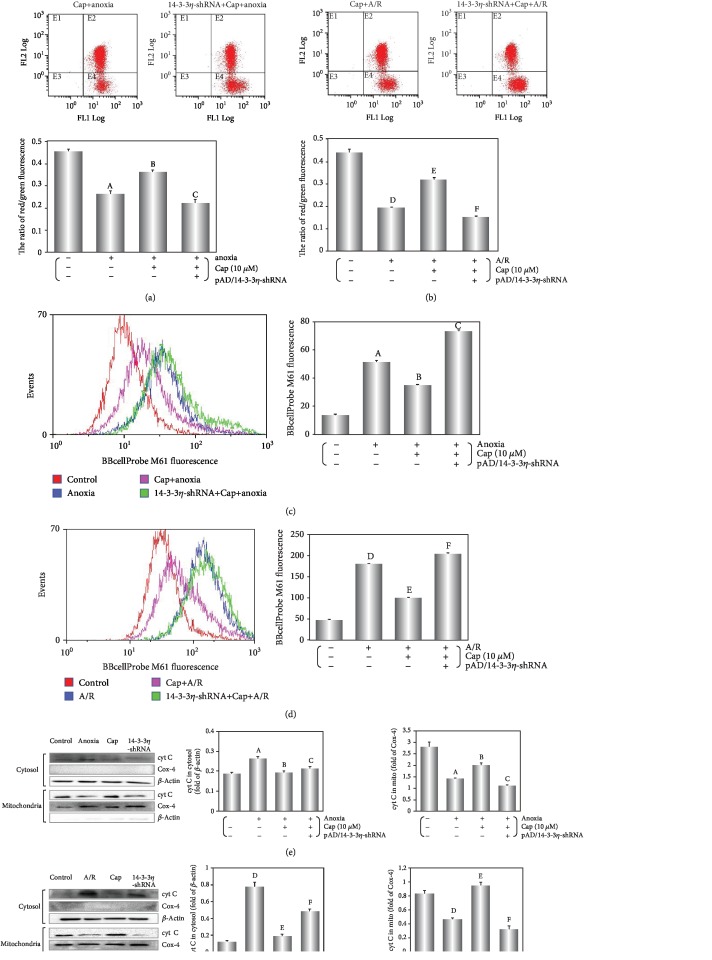
Cap improves mitochondrial function in cardiomyocytes exposed to anoxia or A/R injury. (a, b) Fluorescent dye JC-1 indicating MMP level was detected by flow cytometry, and the ratio of red/green fluorescence is represented. (c, d) Fluorescent probe BBcellProbe M61 indicating mPTP opening was detected by flow cytometry, and column chart of average fluorescence intensity values is shown. (e, f) Western blot and graphic of cyt C level in the cytosol/mitochondria. Data are presented as mean ± SEM (*n* = 6). A: *P* < 0.01 vs. control group (anoxia); B: *P* < 0.01 vs. anoxia group; C: *P* < 0.01 vs. Cap+anoxia group; D: *P* < 0.01 vs. control group (A/R); E: *P* < 0.01 vs. A/R group; F: *P* < 0.01 vs. Cap+A/R group.

A major characteristic of early apoptotic cells is loss of plasma membrane potential [34]. In living cells, the fluorescent dye JC-1 accumulates in the mitochondrial matrix and emits a red fluorescence. However, in apoptotic and dead cells, JC-1 exists as a monomer and emits a green fluorescence. We utilized the red/green fluorescence ratio to express the loss of MMP potential [7]. Both anoxia and A/R exposure induced a loss of MMP (*P* < 0.01) that was rescued by treatment with Cap (*P* < 0.01, Figures 5(a) and 5(b)).

Increased mPTP opening causes the early functional changes of apoptosis [35] with a release of cyt C from mitochondria into the cytosol [36]. As illustrated in Figures 5(c)–5(f), cyt C levels in the cytosol were higher in the A/R group than these in the anoxia alone group (*P* < 0.01), indicating an aggravated mitochondrial malfunction caused by A/R injury. Cap rescue of this effect was significantly stronger in A/R injury stage compared with anoxia alone. As demonstrated in other results, the inhibition of 14-3-3*η* using pAD/14-3-3*η*-shRNA could reverse the effects of Cap (*P* < 0.01).

### 3.5. Cap Decreases Apoptosis of Cardiomyocyte Induced by Anoxia Alone or A/R Injury

**Figure 6:**
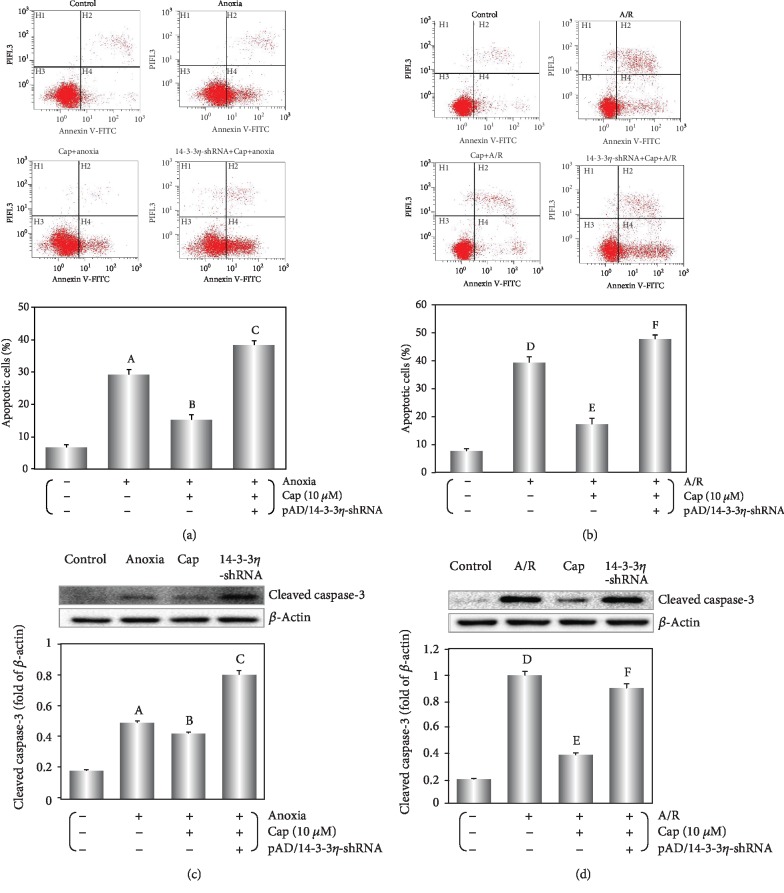
Cap decreases the apoptosis of cardiomyocyte induced by anoxia or A/R. (a, b) Dot plots of Annexin V-FITC/PI detected by flow cytometry and the apoptosis analyzed with the CXP analysis software. (c, d) Western blot and graphic of cleaved caspase-3 levels in cardiomyocytes. Data are presented as mean ± SEM (*n* = 6). A: *P* < 0.01 vs. control group (anoxia); B: *P* < 0.01 vs. anoxia group; C: *P* < 0.01 vs. Cap+anoxia group; D: *P* < 0.01 vs. control group (A/R); E: *P* < 0.01 vs. A/R group; F: *P* < 0.01 vs. Cap+A/R group.

Cleaved caspase-3 is an activated form of caspase-3 [7]. Cleaved caspase-3 expression increased significantly following anoxia or A/R injury (*P* < 0.01, Figures 6(c) and 6(d)). The addition of Cap significantly decreased cleaved caspase-3 expression following injury with anoxia or A/R (*P* < 0.01).

**Figure 7:**
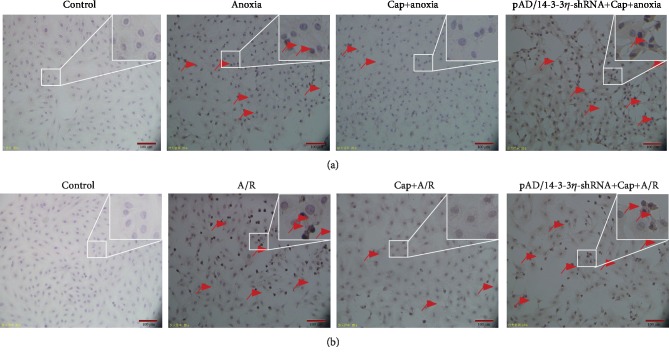
TUNEL assay for apoptotic cells induced by anoxia or A/R injury. Red arrows indicate TUNEL-positive (apoptotic) cardiomyocytes.

Furthermore, apoptosis was measured by flow cytometry [7]. Apoptotic ratio in the anoxia and A/R groups compared with the control group (*P* < 0.01, Figures 6(a) and 6(b)). Cap treatment decreased the apoptotic ratio induced by anoxia or A/R injury (*P* < 0.01). The results of TUNEL staining corroborated the above findings. Varying degrees of accumulation of TUNEL positive cells were identified in anoxia or A/R injury and this was decreased following Cap treatment (Figure 7). Treatment with pAD/14-3-3*η*-shRNA reversed the Cap-mediated rescue of apoptosis (*P* < 0.01).

## 4. Discussion

Anoxia leads to the disorder of energy metabolism in cells, induces oxidative stress, and interferes with the synthesis and function of a large number of proteins [3]. After reoxygenation, cellular function further deteriorates as well studied in cardiomyocyte [24, 25]. In the current study, decreased cell viability and increased LDH and CK activity, cleaved caspase-3 expression, and apoptotic ratio in cardiomyocytes following anoxia or A/R stage (Figures 1, 6, and 7) indicated damage in cardiomyocytes. The impact of A/R injury was stronger compared with anoxia alone in keeping with previously published work [3–9, 24, 25]. Interestingly, Cap significantly blocked the inhibitory effects of anoxia or A/R injury (Figures 1, 6, and 7), suggesting a protective effect on cardiomyocytes following injury, supporting previously published work [7, 8].

As a phytochemical compound with multiple targets and mechanisms of action, Cap regulates the expression and activity of a variety of proteins, further affecting downstream signaling pathways resulting in a biological effect [26, 30]. Cap significantly upregulated 14-3-3*η* and SIRT1 expression, thus promoting translocation of Bcl-2 to mitochondria in cardiomyocytes in response to A/R injury [7, 8]. In this study, we identified that Cap-mediated rescue of cardiomyocytes was linked to 14-3-3*η* expression. This was corroborated by the shRNA-mediated downregulation of 14-3-3*η* expression, which reversed the protective effects of Cap (Figures 1–7).

There are seven known isoforms (*β*, *γ*, *ε*, *η*, *ζ*, *σ*, and *τ*/*θ*) of 14-3-3 family proteins in mammals. Functionally, together with partner proteins, 14-3-3 regulates phosphorylation and dephosphorylation, kinase activity, and cellular location of proteins that may participate in cell proliferation, differentiation, survival, transformation, and apoptosis [37, 38]. Our previous study demonstrated that 14-3-3*η* is activated in ischemia/hypoxia injury while 14-3-3*γ* activation is linked to infectious/inflammatory lesions [7–9, 39]. 14-3-3 is the molecular target of many active ingredients of plants. We have confirmed that 14-3-3 assists PKC*ε*, Bcl-2, and other functional proteins to locate to mitochondria and protect cardiomyocytes and vascular endothelial cells against multiple injuries [7, 9, 40–43]. Further studies are needed to define specific mechanism(s) of action for Cap-activated 14-3-3*η* in anoxia and A/R injured cardiomyocytes.

Mitochondrial dysfunction, a major hallmark of anoxia injury in cardiomyocyte, is exacerbated through reoxygenation to severely affect ROS production and impede cardiomyocyte survival [44, 45]. In this study, we found that ROS generation increased following anoxia alone but was excessive following A/R injury (Figures 3(a) and 3(b)). Mitochondria are furnished with endogenous defense mechanisms against excessive ROS generation [46]. The mechanism of internal defense mainly contains several antioxidant defense systems, among them, GSH/GSSG and NAD^+^/NADH play an important role in maintaining the cellular redox status [47, 48]. Additionally, the balance of ROS and redox states could maintain cellular homeostasis by modulating ion channels, conditioning transports, and regulating enzyme activity [49, 50]. Compared with anoxia alone, GSH/GSSG ratio was dramatically decreased and NAD^+^/NADH ratio was significantly increased by A/R injury. The disruption of antioxidant homeostasis therefore could explain the excessive production of ROS (Figures 3(c)–3(f)).

It is well known that inhibition of activities of mt complexes I and III can result in inducing ROS overproduction and perturbation of the NAD^+^/NADH ratio [51–53]. In the present study, the expression of NDUFB8 and UQCRC2 were decreased in cardiomyocytes after undergoing anoxia or A/R injury (Figures 4(a) and 4(b)). Complexes I/III are inhibited by rotenone and antimycin A, respectively, which leads to the inhibition of the flow of electrons along the respiratory chain and the formation of ATP [54, 55]. Accumulating evidence indicated that the anoxic cardiomyocyte mainly produces ATP by the anaerobic glycolytic pathways [56, 57]. However, accelerated glycolysis of cardiomyocyte in response to impaired pyruvate oxidation could lead to lactate accumulation during anoxia stage [58, 59]. In anoxia or A/R injury, ATP suppression and damaged mitochondrial respiration result from a metabolic flux in cardiomyocytes under pathological conditions (Figures 4(c) and 4(d)). Accordingly, the cardiomyocyte accounted for higher index of glycolytic reserves thus indicating towards mitochondrial malfunction was aggravated during the pathological process (Figures 4(e) and 4(f)). Remarkably, these findings were in keeping with the changes in the redox couples mentioned above, likely because inhibition of mt complexes I/III activity could disturb the redox balance and the homeostasis of cellular energy metabolism during A/R injury.

We found that Cap treatment could increase NADH, GSH, and GSH/GSSG ratio and inhibit NAD^+^, GSSG, and NAD^+^/NADH ratio after anoxia or A/R injury in cardiomyocytes. Contrasted with the results caused by anoxia alone, the doubling ratio of NAD^+^/NADH and GSH/GSSG implied that the ability of Cap to protect cardiomyocyte against external injury was more effective during the A/R period (Figures 3(c)–3(f)). Moreover, Cap treatment could elevate complex I/III expression on cardiomyocyte (Figures 4(a) and 4(b)), increase ATP production-associated mitochondrial respiration, and reduce lactate accumulation (Figures 4(c)–4(f)), while cotreatment with pAD/14-3-3*η*-shRNA could invert effects mentioned above. Therefore, it is difficult to explain the effects as mentioned earlier of Cap just by its antioxidant capacity; the role of Cap upregulating 14-3-3*η* expression and its effects on downstream related pathways are more important. Furthermore, myocardial mitochondrial dysfunction was worse caused by A/R injury than that by anoxia alone. On the contrary, Cap showed a benign protective effect during the gradual deterioration of pathology, which was reflected in redox balance, complexes of ETC, OCR, and ECAR.

Mitochondria are the primary organelle that generates ROS in cardiomyocyte [60]. The excessive ROS generation stimulated MMP and further caused mPTP openness in the inner mitochondrial membrane leading to severe mitochondrial swelling, rupture, and the release of apoptogenic factors [61, 62]. Consistently, pretreatment with Cap stabilized MMP (Figures 5(a) and 5(b)), closed mPTP (Figures 5(c) and 5(d)), and decreased the release of cyt C into the cytoplasm in cardiomyocytes during A/R injury (Figures 5(e) and 5(f)). These responses increased cell viability (Figure 1), decreased cleaved caspase 3 expression (Figures 6(a) and 6(b)), and inhibited apoptosis (Figures 6 and 7).

## 5. Conclusions

Taken together, by comparing the damage from anoxia or reoxygenation, we found that reoxygenation following anoxia could further abrogate the tolerance and adaptability of cardiomyocytes as evidenced by increased ROS generation, inhibited complex I/III activities, and disturbed redox status and homeostasis of cellular energy metabolism. Cap rescued these effects in cardiomyocytes likely through the upregulation of 14-3-3*η*. Cap-treated cardiomyocytes showed improved mitochondrial functioning resulting in apoptosis inhibition.

## Data Availability

The data used to support the findings of this study are included within the article.

## Abbreviations

ANOVA: Analysis of variance
A/R: Anoxia/reoxygenation
ATP: Adenosine triphosphate
BCA: Bicinchoninic acid
Cap: Capsaicin
CK: Creatine kinase
cyt C: Cytochrome C
DAB: Diaminobenzidine
DCFH-DA: 6-Carboxy-2′-7′-dichlorodihydro-fluorescein diacetate
DMEM: Dulbecco's modified Eagle medium
ECAR: Extracellular acidification rate
ETC: Electron transport chain
FBS: Fetal bovine serum
GSH: Reduced glutathione
GSSG: Oxidized glutathione
HBSS: Hank's balanced salt solution
I/R: Ischemia/reperfusion
JC-1: 5,5′,6,6′-Tetrachloro-1,1′,3,3′-tetraethyl-benzimidazolo carbocyanine iodide
LDH: Lactate dehydrogenase
LSD: Least significant difference
mPTP: Mitochondrial permeability transition pore
MMP: Mitochondrial membrane potential
NAD^+^: Oxidized nicotinamide adenine dinucleotide
NADH: Reduced nicotinamide adenine dinucleotide
NDUFB8: NADH dehydrogenase [ubiquinone] 1 beta subcomplex subunit 8
OCR: Oxygen consumption rate
PBS: Phosphate-buffered saline
PI: Propidium iodide
PMSF: Phenylmethanesulfonyl fluoride
ROS: Reactive oxygen species
SEM: Standard error of mean
TUNEL: Terminal deoxynucleotidyl transferase dUTP nick-end labeling
UQCRC2: Cytochrome b-c1 complex subunit 2
WST-8: 2-(2-Methoxy-4-nitrophenyl)-3-(4-nitrophenyl)-5-(2,4-disulfophenyl)-2H-tetrazolium, monosodium salt.
